# Perception of risk of hypertension related complications and adherence to antihypertensive drugs: a primary healthcare based cross-sectional study

**DOI:** 10.1186/s12875-022-01918-1

**Published:** 2022-11-29

**Authors:** Ramin Shiraly, Ali Khani Jeihooni, Rozita Bakhshizadeh Shirazi

**Affiliations:** 1 Department of Community Medicine, School of Medicine, Health Behavior Science Research Center, Shiraz University of Medical Sciences, Shiraz, Iran; 2 Nutrition Research Center, Department of Public Health, School of Health, Shiraz University of Medical Sciences, Shiraz, Iran; 3 Zarghan Hospital, Shiraz University of Medical Sciences, Shiraz, Iran

**Keywords:** Hypertension, Complications, Perceived risk, Medication adherence

## Abstract

**Background:**

Blood pressure control is suboptimal in more than half of treated hypertensive patients. The purpose of this study was to assess perceived risk of high blood pressure sequelae and adherence to medications in known cases of hypertension.

**Methods:**

A cross-sectional study was designed using a sample of 600 hypertensive patients who were randomly selected from 32 primary healthcare centers in Shiraz, Iran. A structured interviewer-administered questionnaire was used to collect data. Participants were asked about their basic demographic information, smoking history, access to healthcare services, duration of antihypertensive therapy, number of drugs taken concurrently and their perceived risk of hypertension-related complications. The outcome of interest was adherence to antihypertensive medications measured by the Persian version of the Morisky Medication Adherence Scale (MMAS-8). Multivariate logistic regression analysis was used to identify independent factors associated with better adherence.

**Results:**

Nearly half (48.8%) of participants had uncontrolled hypertension. Just over one fifth (22.3%) of all the patients reported high adherence to antihypertensive medications. Independent factors associated with better adherence to antihypertensive medications were higher educational level (OR: 1.71, CI 95%: 1.06–2.75), being a never smoker (OR: 1.62, CI 95%: 1.06–2.46), having easy access to healthcare services (OR: 1.91, CI 95%: 1.10–3.35), lower mean treatment duration (OR: 0.96, CI 95%: 0.92–0.99), and having higher perceived risk of hypertension-related complications (OR:2.34, CI 95%: 1.52–3.60).

**Conclusion:**

High perceived risk of hypertension-related complications is significantly associated with adherence to antihypertensive therapy. Our findings suggest that primary care physicians should regularly emphasize on negative consequences of uncontrolled/poorly controlled blood pressure while visiting hypertensive patients.

## Introduction

Hypertension (HTN) is the principal risk factor for cardiovascular diseases and the leading cause of premature death worldwide. Global prevalence of HTN in adults vary by age, gender and country-level income and ranges from 25 to 39% [1]. Estimated trends of raised blood pressure (BP) across the world during the last decades suggest that there has been a shift in prevalence of high BP from high- income countries to low- and middle-income countries. While the rates of awareness, treatment and control of HTN have markedly improved in high-income countries, these rates have slightly improved or remained unchanged in most middle- and low-income countries [2–4].

Untreated or uncontrolled HTN increases the risk of stroke, heart failure, coronary artery disease, and kidney disease [5]. Despite extensive screening and educational programs and availability of lots of antihypertensive (AH) drug regimens, the proportion of treated hypertensive patients with controlled BP is still suboptimal, even in highly developed countries. Population-based studies from United States, Australia and Southern America reported that at least 40% of treated hypertensive patients had uncontrolled BP [6, 7]. Although several factors including demographic and socioeconomic determinants, optimal drug choice and dosing, frequent monitoring of blood pressure and contact with healthcare system are associated with improved HTN management, consistent adherence to pharmacotherapy is a key factor in achieving BP control and good clinical outcomes in hypertensive patients [8].

Medication adherence is defined by the World Health Organization (WHO) as “the extent to which the person’s medication-taking behavior corresponds with the agreed recommendations from a health care provider [9]. Poor medication adherence is an important public health issue, particularly for patients with chronic diseases and can lead to poor health outcomes and increased costs of healthcare [10]. Medication adherence can be associated with multiple factors at the drug, patient, provider and healthcare setting levels [11].

In spite of numerous studies on factors affecting adherence to antihypertensive medications, very little of the research has focused on perceived risk of hypertension sequelae as a determinant of patients’ compliance with therapy. The main aim of the present study was to evaluate risk perception of complications of hypertension as a potential influencing factor for treatment adherence among a sample of primary care patients with known hypertension in Shiraz, Southwestern Iran. We hypothesized that those with high risk perception are more likely to adhere to antihypertensive medications. This hypothesis was based on the belief that every health decision-making is at least partly driven by an individual’s risk perception of the potential positive and negative outcomes associated with doing or not doing a specific behavior [12]. .Extensive experimental scientific investigations have provided convincing evidence that interventions targeting risk perception can positively influence patients’ health-related behaviors [13]. Therefore, addressing this issue may suggest strategies to improve hypertensive patients’ treatment adherence and thus their health outcomes.

## Methods

### Study setting and participants

We conducted a cross-sectional study of adults who had a known diagnosis of HTN and were receiving health services at the primary healthcare (PHC) centers of Shiraz, Southwestern Iran. The inclusion criteria were being Iranian, aged 30 years and older, and being diagnosed with essential hypertension and were receiving AH medications for at least 12 months. Participants were recruited using random cluster sampling method. The sampling approach was based on PHC centers, taking into account the geographical distribution of centers representing different municipal areas and socioeconomic backgrounds. In the first stage, all the PHC centers of the city were numbered and 32 (out of 68) centers were randomly selected. In the second stage, in each selected PHC center, a list of eligible hypertensive patients was generated through the national electronic health information system for Iranian population. The target sample size for the present study was 600 patients. Twenty to thirty patients were randomly selected from the list at each center and were invited to participate in the study by telephone. The enrollment process was continued until the required sample size was attained. Eligible patients who accepted our invitation took part in a face-to-face interview. Before starting the interview, participants were given a verbal explanation about the objectives of the study and completed an informed consent form. After the interview, patients who were identified as non-compliant with treatment or had uncontrolled BP were referred to the physician for further evaluation. This study was reviewed and approved by Research Ethics Committee of Shiraz University of Medical Sciences (IR.sums.med.rec.1396.s153).

### Measurements

Basic demographic information included age, gender, marital and working status, educational level and urban/rural residence.

To evaluate cigarette smoking status of participants, smoking habits were classified as current smoker, ex-smoker and never smoker. Current smoker was defined as a person who had regularly smoked in the past month. An ex-smoker was defined as person who had regularly smoked but had not smoked in the past month. A never smoker was defined as someone who has never smoked cigarettes.

Access to healthcare was assessed by a single item question: “Do you have easy access to healthcare services?” This measure was used to assess participants’ ability to reach, pay and obtain healthcare services when they get sick. The accessibility of healthcare is a multidimensional concept which may be affected by different factors such as approachability, availability, affordability and appropriateness of healthcare services [14].

Medication-related factors included AH treatment duration and complexity of medication regimen, including number of daily doses and concurrent medications. When two or more AH drugs were administered in one tablet or capsule (fixed-dose combination therapy), the drug was considered as a single medication.

History of cardiovascular diseases / events was defined as individuals who reported having been ever diagnosed with coronary heart disease (CHD), myocardial infarction (MI) or stroke.

Perceived risk of complications of high BP was assessed using three questions. The questions were adapted from a previous study conducted by Cummings et al. [15] and were discussed and verified in the research team. The items were finally reviewed and validated by two expert epidemiologists. Respondents were asked to rate their risk of developing myocardial infarction, stroke and chronic kidney disease (CKD) due to complications of high blood pressure during future 10 years. Responses were rated as 5 part Likert-type scale from very low (1) to very high (5). Items were summed to create a total perceived risk score that ranged from 3 to 15, a higher score indicating higher perceived risk. Internal consistency between the three items was acceptable (Cronbach’s α = 0.89).

We used the Persian version of the Morisky Medication Adherence Scale (MMAS-8) to assess adherence to antihypertensive medications [16]. MMAS-8 is commonly regarded as the one of the most valid self-report means of evaluating adherence to medication [9]. This 8-item scale evaluates medication taking behaviors and is able to identify medication non-adherence and barriers to adherence. The scale contributes to better understand the patients’ medication taking behaviors including those that might be unintentional (e.g., forgetfulness) or intentional (e.g., discontinuing medications because of adverse effects) [17]. The first seven items are responded to through no/yes options and are scored 1 for adherent or 0 for non-adherent behavior, except for Q5 (did you take your medications yesterday?) which is scored in the reverse order. The item 8 assesses how often patients forget to take their medications using a 5-point Likert responses which are rated from 0 (All the time) to 1 (Never/rarely). Scores from individual items are summed to produce an overall adherence score ranging from 0 to 8, with higher scores representing better medication adherence. Total scores have been categorized into three levels of adherence: high adherence (score = 8), medium adherence (score of 6 to 7), and low adherence (score < 6) [18]. Cronbach’s alpha in the present study was 0.715.

### Statistical analysis

Demographic factors were described as frequency and percentage for categorical variables and mean and standard deviation for numerical variables. Chi-square test and the Kruskal Wallis test was used to detect statistical differences between patients with different levels of medication adherence. The spearman rank correlation test was used to estimate the strength of possible associations between predictive and outcome variables and to examine collinearity between numerical independent variables. Multivariate logistic regression were established to determine independent variables associated with high adherence to AH drugs. When building the multivariate models, each of the independent variables were evaluated in univariate models, and those with *p*-values < 0.10 were considered for inclusion in the multivariate logistic regression. The effects of age was controlled in the regression model. SPSS version 28 (IBM, United States) was used for all statistical analysis and significance level was set at 5%.

## Results

Among 600 patients who were interviewed, there were 303 women (50.5%) and 297 men (49.5%). The mean age of participants was 59.3 years. Of the 600 participants, 134 (22.3%) had high adherence and 117 (19.5%) reported low adherence to AH medications. The MMAS-8 score was significantly associated with BP control (*P* <  0.001). The mean MMAS-8 score was significantly higher among patients with controlled BP (6.7) than those with uncontrolled BP (6.0) (*P* <  0.001). Table 1 shows the characteristics for hypertensive patients with different levels of drug adherence. Patients with high drug adherence, compared to those with low adherence, were significantly more likely to (i) have a lower mean age (57 vs. 60), (ii) have a college degree (28% vs. 14%), (iii) be a never smoker (63% vs. 39%), (iv) report not having a history of cardiovascular disease / events (72% vs. 54%), (v) report lower mean years of AH treatment (6 vs. 8), (vi) report not taking more than two AH drugs concurrently (93% vs. 81%), (vii) have easy access to healthcare services (87% vs 71%), (viii) have higher mean perceived risk score of HTN-associated MI (3.4 vs. 2.6), stroke (3.7 vs. 2.9) and CKD (4 vs. 3).

**Table 1:** Characteristics of study participants (*n* = 600)

Characteristic	High adherence (*n* = 134) N (%)	Moderate adherence (*n* = 349) N (%)	Low adherence (*n* = 117) N (%)
**Demographics**			
Age, mean (SD)	56.7 ± 10.8	60.1 ± 11.6	59.6 ± 12.1^**^
Gender			
*Male*	59 (44)	180 (51.6)	58 (49.6)
*Female*	75 (56)	169 (48.4)	59 (50.4)
Residence			
*Urban*	72 (53.7)	166 (47.6)	62 (53)
*Rural*	62 (46.3)	183 (52.4)	55 (47)
Marital status			
*Married*	111 (82.8)	284 (81.4)	91 (77.8)
*Single/Divorced/Widowed*	23 (17.2)	65 (18.6)	26 (22.2)
Work status			
*Working*	34 (25.4)	76 (21.8)	27 (23.1)
*Not working*	100 (74.6)	273 (78.2)	90 (76.9)
Educational level			
*Illiterate / Primary school*	71 (53)	226 (64.8)	84 (71.8)^*^
*High school*	26 (19.4)	62 (17.8)	17 (14.5)
*College*	37 (27.6)	61 (17.5)	16 (13.7)
**Smoking status**			
*Current smoker*	25 (18.7)	98 (28.1)	33 (28.2)^**^
*Ex- smoker*	25 (18.7)	91 (26.1)	38 (32.5)
*Never smoker*	84 (62.6)	160 (45.8)	46 (39.3)
**Blood pressure status**^**a**^			
*Uncontrolled (≥ 140/90)*	53 (39.6)	158 (45.3)	82 (70)^***^
*Controlled (< 140/90)*	81 (60.4)	191 (54.7)	35 (30)
**History of cardiovascular events**			
*No*	96 (71.6)	213 (61)	63 (53.8)^*^
*Yes*	38 (28.4)	136 (39)	54 (46.2)
**Medication-taking status**			
Years of AH drug therapy, mean (SD)	5.6 ± 4.3	7.8 ± 6.4	8.2 ± 6.6^***^
No. of AH drugs taken concurrently			
*1*	71 (53)	142 (40.7)	52 (44.4)^**^
*2*	54 (40.3)	170 (48.7)	43 (36.8)
*≥ 3*	9 (6.7)	37 (10.6)	22 (18.8)
Frequency of AH drug use per day			
*once*	78 (58.2)	167 (48)	51 (43.6)
*2 times*	48 (35.8)	35 (42)	58 (49.6)
*3–4 times*	8 (6)	35 (10)	8 (6.8)
**Access to healthcare**			
*Easy*	116 (86.6)	272 (77.9)	83 (71)^*^
*Difficult*	18 (13.4)	77 (22.1)	34 (29)
**Perceived risk score, mean (SD)**	11.1 ± 2.9	9.5 ± 3.3	8.6 ± 3.8^***^

^a^Patient’s blood pressure measurement at the time of study

^*^P <  0.05

^**^P <  0.01

^***^P <  0.001

Results of correlations between MMAS-8 scores & numerical study variables are shown in Table 2. According to the Spearman rank correlation coefficient, there was a negative relationship between mean years of AH therapy and MMAS-score (r = − 0.115, *p* = 0.005). Also, there was a positive correlation between patients’ perceived risk of HTN-related complications and MMAS-8 scores (r: 0.219; *P* <  0.001), indicating that higher levels of perceived risk is associated with better adherence to AH medications.

**Table 2:** Correlation between age, duration of antihypertensive therapy (in years), Perceived risk score for hypertension sequelae and MMAS-8 scores among participant patients

Correlation variables	r^a^	*P* value
MMAS-8 score^b^ / Age	- 0.095	0.02
MMAS-8 score / Duration of therapy	- 0.115	0.005^*^
MMAS-8 score / Perceived risk score	0.219	< 0.001^*^
Perceived risk score / Age	- 0.294	< 0.001^*^
Perceived risk score / Duration of therapy	- 0.367	< 0.001^*^
Age / Duration of therapy	0.564	< 0.001^*^

^a^Spearman’s correlation coefficient

^b^Higher MMAS-8 scores indicate better medication adherence

^*^Correlation is significant at the 0.01 level (2-tailed)

Table 3 shows the association between study variables and the levels of drug adherence. Independent factors associated with better AH medication adherence among Iranian hypertensive patients were higher educational level (OR: 1.71, CI 95%: 1.06–2.75), being a never smoker (OR: 1.62, CI 95%: 1.06–2.46), having easy access to healthcare services (OR: 1.91, CI 95%: 1.10–3.35), lower mean AH treatment duration (OR: 0.96, CI 95%: 0.92–0.99), and having higher perceived risk of HTN-related complications (OR:2.34, CI 95%: 1.52–3.60).

**Table 3:** Factors associated with adherence to antihypertensive medications

Factors	Univariate model		Multivariate model	
	OR (95% CI)	***p***-value	OR (95% CI)	***p***-value
Higher age	0.98 (0.96–0.99)	0.004	1.01 (0.99–1.03)	0.360
Female sex	1.33 (0.90–1.95)	0.151	–	–
Married status	1.17 (0.71–1.94)	0.539	–	–
Not working	0.84 (0.53–1.30)	0.427	–	–
Higher educational level	1.93 (1.23–3.03)	0.004	1.71 (1.06–2.75)	0.028^*^
Urban residence	1.21 (0.83–1.78)	0.378	–	–
Never smoker	2.12 (1.43–3.15)	< 0.001	1.62 (1.06–2.46)	0.025^*^
Cumulative years of AH therapy	0.92 (0.89–0.96)	< 0.001	0.96 (0.92–0.99)	0.047^*^
Lower no. of medications taken per day	2.01 (0.97–4.17)	0.063	1.04 (0.46–2.37)	0.909
More frequent drug use per day	0.63 (0.29–1.36)	0.237	–	–
History of cardiovascular events	1.74 (1.14–2.64)	0.009	1.24 (0.77–1.99)	0.373
Easy access to healthcare services	2.01 (1.17–3.46)	0.011	1.91 (1.10–3.35)	0.024^*^
High perceived risk of hypertension sequelae	3.10 (2.05–4.58)	< 0.001	2.34 (1.52–3.60)	< 0.001^*^

^*^Statistically significant

## Discussion

Our study showed a significant association between perceived risk of HTN sequelae and adherence to AH medications in a primary care-based sample of patients with diagnosed hypertension. In addition, we found that patients with low medication adherence were more likely to have uncontrolled BP than those with high adherence (70.1% vs. 39.6%, *P* <  0.001). These findings suggest that perception of seriousness of the disease may play an important role in predicting BP control in hypertensive patients. Previous studies reported that poor medication adherence could contribute to inadequate BP control in as much as two thirds of hypertensive patients and a prospective study showed that behavioral interventions targeting perceived risk of complications of poorly controlled BP could improve adherence to AH medications [19]. Similarly, a study conducted in rural India found that patients who perceived high susceptibility to HTN, had better adherence to AH therapy [20]. Considering the relationship between medication adherence and BP control and given the asymptomatic nature of hypertension, it seems that the risk perception of the disease sequelae may motivate patients to remain on treatment.

Our results further confirmed an inverse relationship between number of drugs taken concurrently and levels of medication adherence. In this respect, there is significant evidence that complexity of drug regimen can negatively affect adherence and, therefore, reducing the number of medications and the frequency of administration would result in better compliance with treatment [9, 21].

In univariate analysis, there was no statistically significant differences between the participants’ levels of medication adherence in terms of demographic variables, except for age and educational level. However, after conducting a multivariable logistic regression analysis, education was the only significantly associated demographic factor, with higher educational level positively associated with medication adherence.

Based on our findings, there is a less clear evidence of an association between AH treatment duration and adherence to AH medications, as former studies have provided different results when assessing relations between patients’ age, duration of use and adherence to AH medications [22]. Reports from middle- and low-income countries have shown that older age predicts better adherence to AH medications [23]. In contrast, in a study on 1000 Greek hypertensive patients, adherence to medications was positively associated with younger age (age < 60 years) [24]. In our study, the association between duration of AH therapy and medication adherence was significant and remained significant after controlling for age. Regardless of kind of disease, there has been some evidence that adherence to treatment decreases over time [25].

Our findings also showed that low adherence to AH medications was associated with being a current or ex-smoker. A previous study on a large sample of Japanese patients with non-communicable chronic disease indicated that medication non-adherence was significantly and positively associated with current smoking habits [26].

In summary, we found a positive association between patients’ perceived risk of complications and compliance with AH therapy. Based on our literature review, little studies have investigated this factor as an independent determinant of medication adherence. As a general concept, it appears that patients’ medication adherence is more strongly influenced by treatment-related beliefs and perceptions than socio-demographic factors (education, gender, age) and clinical variables (type of illness, number of medications) [27]. Indeed, beliefs that negligence to take medicine could result in unfavorable outcomes, tends to be associated with higher adherence rates [28]. We believe that adherence to AH medications is, likewise, a behavior that is largely influenced by personal psychological feelings and perceptions toward disease and treatment. As an influencing modifiable factor, patients’ perceived risk of HTN-related complications should be addressed and targeted when designing interventions to improve adherence to AH medications.

There are some limitations to our study that should be noted. This research was a cross-sectional study based on patients’ self-reports and does not provide strong evidence about associations. The study participants were recruited from PHC centers in Shiraz and might not be representative of all Iranian hypertensive patients. We did not evaluate some factors which have been suggested as consistent factors associated with medication non-adherence such as cost-related problems, medication side effects and poor patient-provider communication [29]. Most of these factors raise the risk of unintentional non-adherence and may not be directly linked to patients^,^ perceptions and attitudes toward the risk and benefits of the treatment [9].

## Conclusion

High perceived risk of hypertension-related complications is significantly associated with adherence to antihypertensive therapy. Patients must be cognizant of complications of the high BP, as this may improve their adherence to AH therapy. It is recommended that physicians should regularly emphasize on negative consequences of uncontrolled/poorly controlled BP while visiting hypertensive patients to enhance their perceived risk, particularly at the primary care level.

## Data Availability

The datasets generated and/or analyzed during the current study are not publicly available due to due to institutional regulations and privacy restrictions but are available from the corresponding author upon reasonable request.

## Abbreviations

HTN: Hypertension
BP: Blood pressure
AH: Antihypertensive
PHC: Primary healthcare center
OR: Odds Ratio

## References

[1] Mills KT, Stefanescu A, He J (2020). The global epidemiology of hypertension. Nat Rev Nephrol.

[2] NCD Risk Factor Collaboration (NCD-RisC) (2017). Worldwide trends in blood pressure from 1975 to 2015: a pooled analysis of 1479 population-based measurement studies with 19·1 million participants. Lancet..

[3] McAlister FA, Wilkins K, Joffres M, Leenen FH, Fodor G, Gee M, Tremblay MS, Walker R, Johansen H, Campbell N (2011). Changes in the rates of awareness, treatment and control of hypertension in Canada over the past two decades. CMAJ.

[4] Kang SH, Kim SH, Cho JH, Yoon CH, Hwang SS, Lee HY, Youn TJ, Chae IH, Kim CH (2019). Prevalence, awareness, treatment, and control of hypertension in Korea. Sci Rep.

[5] Fisher NDL, Curfman G (2018). Hypertension-a public health challenge of global proportions. JAMA..

[6] Chowdhury EK, Nelson MR, Ernst ME, Margolis KL, Beilin LJ, Johnston CI (2020). Factors associated with treatment and control of hypertension in a healthy elderly population free of cardiovascular disease: a cross-sectional study. Am J Hypertens.

[7] Rubinstein AL, Irazola VE, Calandrelli M, Chen CS, Gutierrez L, Lanas F, Manfredi JA, Mores N, Poggio R, Ponzo J, Seron P, Bazzano LA, He J (2016). Prevalence, awareness, treatment, and control of hypertension in the southern cone of Latin America. Am J Hypertens.

[8] Burnier M, Egan BM (2019). Adherence in hypertension. Circ Res.

[9] Jimmy B, Jose J (2011). Patient medication adherence: measures in daily practice. Oman Med J.

[10] Lam WY, Fresco P (2015). Medication adherence measures: an overview. Biomed Res Int.

[11] Smaje A, Weston-Clark M, Raj R, Orlu M, Davis D, Rawle M (2018). Factors associated with medication adherence in older patients: a systematic review. Aging Med (Milton).

[12] Ferrer R, Klein WM (2015). Risk perceptions and health behavior. Curr Opin Psychol.

[13] Sheeran P, Harris PR, Epton T (2014). Does heightening risk appraisals change people's intentions and behavior? A meta-analysis of experimental studies. Psychol Bull.

[14] Cyr ME, Etchin AG, Guthrie BJ, Benneyan JC (2019). Access to specialty healthcare in urban versus rural US populations: a systematic literature review. BMC Health Serv Res.

[15] Cummings KM, Kirscht JP, Binder LR, Godley AJ (1982). Determinants of drug treatment maintenance among hypertensive persons in inner city Detroit. Public Health Rep.

[16] Moharamzad Y, Saadat H, Nakhjavan Shahraki B (2015). Validation of the Persian version of the 8-item Morisky medication adherence scale (MMAS-8) in Iranian hypertensive patients. Global J Health Sci.

[17] De Las CC, Peñate W (2015). Psychometric properties of the eight-item Morisky medication adherence scale (MMAS-8) in a psychiatric outpatient setting. Int J Clin Health Psychol.

[18] Morisky DE, Ang A, Krousel-Wood M, Ward HJ (2008). Predictive validity of a medication adherence measure in an outpatient setting. J Clin Hypertens (Greenwich).

[19] Bosworth HB, Olsen MK, Neary A, Orr M, Grubber J, Svetkey L, Adams M, Oddone EZ (2008). Take control of your blood pressure (TCYB) study: a multifactorial tailored behavioral and educational intervention for achieving blood pressure control. Patient Educ Couns.

[20] Venkatachalam J, Abrahm SB, Singh Z, Stalin P, Sathya GR (2015). Determinants of Patient's adherence to hypertension medications in a rural population of Kancheepuram District in Tamil Nadu, South India. Indian J Community Med.

[21] Ingersoll KS, Cohen J (2008). The impact of medication regimen factors on adherence to chronic treatment: a review of literature. J Behav Med.

[22] Assawasuwannakit P, Braund R, Duffull SB (2015). A model-based meta-analysis of the influence of factors that impact adherence to medications. J Clin Pharm Ther.

[23] Nielsen JØ, Shrestha AD, Neupane D, Kallestrup P (2017). Non-adherence to anti-hypertensive medication in low- and middle-income countries: a systematic review and meta-analysis of 92443 subjects. J Hum Hypertens.

[24] Yiannakopoulou EC, Papadopulos JS, Cokkinos DV, Mountokalakis TD (2005). Adherence to antihypertensive treatment: a critical factor for blood pressure control. Eur J Cardiovasc Prev Rehabil.

[25] Kjellström B, Sandqvist A, Hjalmarsson C, Nisell M, Näsman P, Ivarsson B (2020). Adherence to disease-specific drug treatment among patients with pulmonary arterial hypertension or chronic thromboembolic pulmonary hypertension. ERJ Open Res.

[26] Nakajima R, Watanabe F, Kamei M (2021). Factors associated with medication non-adherence among patients with lifestyle-related non-communicable diseases. Pharmacy (Basel).

[27] Horne R, Buick D, Fisher M, Leake H, Cooper V, Weinman J (2004). Doubts about necessity and concerns about adverse effects: identifying the types of beliefs that are associated with non-adherence to HAART. Int J STD AIDS.

[28] Horne R, Weinman J, Hankins M (1999). The beliefs about medicines questionnaire: the development and evaluation of a new method for assessing the cognitive representation of medication. Psychol Health.

[29] van der Laan DM, Elders PJM, Boons CCLM, Beckeringh JJ, Nijpels G, Hugtenburg JG (2017). Factors associated with antihypertensive medication non-adherence: a systematic review. J Hum Hypertens.

